# Altered Methylation of *IGF2* Locus 20 Years after Preterm Birth at Very Low Birth Weight

**DOI:** 10.1371/journal.pone.0067379

**Published:** 2013-06-19

**Authors:** Karoliina Wehkalampi, Mari Muurinen, Sara Bruce Wirta, Katariina Hannula-Jouppi, Petteri Hovi, Anna-Liisa Järvenpää, Johan G. Eriksson, Sture Andersson, Juha Kere, Eero Kajantie

**Affiliations:** 1 Department of Health Promotion and Chronic Disease Prevention, National Institute for Health and Welfare, Helsinki, Finland; 2 Children’s Hospital, Helsinki University Central Hospital, Helsinki, Finland; 3 Unit of General Practice, Helsinki University Central Hospital, Helsinki, Finland; 4 Skin and Allergy Hospital, Helsinki University Central Hospital, Helsinki, Finland; 5 Department of General Practice and Primary Health Care, University of Helsinki, Helsinki, Finland; 6 Research Programs Unit, Molecular Medicine Program, University of Helsinki, Helsinki, Finland; 7 Folkhälsan Institute of Genetics, University of Helsinki, Helsinki, Finland; 8 Folkhälsan Research Center, University of Helsinki, Helsinki, Finland; 9 Department of Biosciences and Nutrition, Center for Biosciences, Karolinska Institute, Stockholm, Sweden; Johns Hopkins University School of Medicine, United States of America

## Abstract

**Introduction:**

People born preterm at very low birth weight (VLBW, ≤1500g) have higher rates of risk factors for adult-onset diseases, including cardiovascular diseases and type 2 diabetes. These risks may be mediated through epigenetic modification of genes that are critical to normal growth and development.

**Methods:**

We measured the methylation level of an imprinted insulin-like-growth-factor 2 (*IGF2*) locus (*IGF2/H19*) in young adults born preterm at VLBW and in their peers born at term. We studied 158 VLBW and 161 control subjects aged 18 to 27 years from the Helsinki Study of Very Low Birth Weight Adults. Methylation fraction at two *IGF2* differentially methylated regions (DMRs) – *IGF2* antisense transcript (*IGF2AS,* also known as *IGF2* DMR0) and last exon of *IGF2* (*IGF2_05*, also known as *IGF2* DMR2) – were measured with Sequenom Epityper. We used linear regression and adjustment for covariates to compare methylation fractions at these DMRs between VLBW and control subjects.

**Results:**

At one *IGF2AS* CpG site, methylation was significantly lower in VLBW than in control subjects, mean difference −0.017 (95% CI; −0.028, −0.005), P = 0.004. Methylation at *IGF2_05* was not different between the groups.

**Conclusions:**

Methylation of *IGF2AS* is altered 20 years after preterm birth at VLBW. Altered methylation may be a mechanism of later increased disease risk but more data are needed to indicate causality.

## Introduction

It has been hypothesized that many complex adult chronic diseases originate from conditions encountered *in utero* and in early life. This was first perceived in people born at term with a lower birth weight who have higher rates of many adult-onset diseases (cardiovascular diseases and type 2 diabetes) or their risk factors [1]–[4]. Also individuals that were *in utero* exposed to caloric deprivation during the Dutch and Biafran famines have been shown to be in excess risk of several common chronic diseases in adulthood [5], [6]. Similarly people born preterm at very low birth weight (VLBW, birth weight ≤1500 g) encounter risks for late-onset chronic diseases [7]–[12]. For instance, in comparison to their non-low-birth-weight term-born peers, VLBW subjects have higher blood pressure [7]–[10] as well as impaired glucose regulation [11], [12].

Individuals born at term with a low birth weight, exposed to famine *in utero* or born preterm at VLBW all share a period of poor nutrition and growth early in life [13]–[15]. In the Dutch famine studies, early caloric deprivation has been shown to be associated with aberrant methylation at the Insulin-like growth factor-2 (*IGF2*) gene [16], [17], which is an imprinted gene that functions as part of the anabolic growth hormone/insulin-like growth factor endocrine axis regulating growth. Imprinted genes are epigenetically labile to early environmental influences [18] and modest methylation shifts in their functional/regulatory regions are associated with changes in gene expression. *IGF2* is particularly critical for growth in fetal life and plays a role in fetal growth restriction [19]. Whether a change in *IGF2* methylation results in enhanced incidence of chronic diseases in the Dutch famine study population is presently unknown. However, evidence for its role as a link between early conditions and later adult disease risk comes from several other studies reporting associations between methylation shifts at *IGF2* differentially methylated regions (DMRs) and increased susceptibility to e.g. malignancies, as well as obesity [20]. Variation in *IGF2* methylation could potentially serve as a mediator between adult cardiovascular disease (CVD) risk and preterm birth at VLBW.

In this study we investigated the level of *IGF2* (*IGF2/H19* locus) methylation in a population of young adults born preterm at VLBW, in whom we previously have reported elevated levels of CVD risk factors – higher blood pressure and impaired glucose regulation [10], [12] – in comparison to their non-low-birth-weight term-born controls. We chose to study hierarchically regulated *IGF2* DMRs through which imprinting of the *IGF2* gene is maintained. The first DMR we chose was the *IGF2* antisense transcript (*IGF2AS)* amplicon, also known as *IGF2* DMR0 [21], in which both gain and loss of methylation have been reported under different conditions [16], [17]. The other amplicon we chose to study was *IGF2*-DMR2, overlapping the last exon of *IGF2* (*IGF2_05*) [22].

### Subjects

The participants of this study come from The Helsinki Study of Very Low Birth Weight Adults, which is a longitudinal follow-up cohort of subjects born preterm at VLBW between 1978 and 1985 and treated in the Neonatal Intensive Care Unit of Children's Hospital of Helsinki University Central Hospital. Of the original cohort, 255 VLBW subjects had survived from the intensive care period and resided in the greater Helsinki area at the time of invitation for follow-up. These subjects were invited to participate in the study together with a sex-, age-, and birth hospital -matched comparison group of 314 term-born subjects who were not small for gestational age (SGA), i.e. whose birth weight was more than –2 SD, based on Finnish growth standards [23]. Clinical visits of the study participants as well as non-participant analysis are explained in more detail elsewhere [10], [12], [24]. Altogether, 165 (65%) VLBW and 172 (55%) control subjects participated in a clinical examination in their early adulthood, at age 18 to 27 years. The examination included, e.g., height and weight measurement based on which body mass index (BMI) was calculated (weight in kilograms divided by height in meters squared), and a 2-hour oral glucose tolerance test. The subjects also completed detailed questionnaires that covered their medical history, lifestyle factors, and familial history of disease. Furthermore, blood samples were drawn for epigenetic analyses (*IGF2* methylation fraction measurement). These samples were taken in a fasting state and blood was stored as EDTA blood in a freezer (–20°C). For eight VLBW and 11 control subjects the *IGF2* methylation fraction data did not pass quality control. We obtained reliable methylation fractions for 158 VLBW and 161 controls. For 127 VLBWs and 146 controls, we also carried out dual-energy x-ray absorptiometry (DXA, Discovery A, Hologic) to measure body composition.

This study was performed according to the declaration of Helsinki. The study protocol was approved by the Ethics committee of the Helsinki and Uusimaa Hospital District, Finland. Written informed consent was obtained from each participant.

## Methods

### Assessment of methylation fractions

In the two DMRs, *IGF2AS* and *IGF2_05,* we measured the methylation level of CpG units (i.e. fragments containing one or more CpG sites) – six units for *IGF2AS* and seven units for *IGF2_05 –* using Sequenom EpiTYPER (Sequenom, San Diego, CA, USA). A fragment is produced from a PCR amplicon, designed to contain at least 4 analyzable CpGs, which is initially amplified from bisulfite treated DNA. The PCR amplicon is subsequently transcribed in vitro and fragmented by RNAase to produce smaller fragments where the cytosines of unmethylated CpGs are present as adenosines and of methylated CpGs as guanines. These fragments differ in mass which the EpiTYPER software identifies. Genomic DNA was extracted from whole peripheral EDTA blood by using QIAamp DNA Blood Maxi Kit (Qiagen®); 500 ng of DNA was then bisulfite-treated using the EZ DNA Methylation Kit (Zymo Research Corporation, Irvine, CA, USA) according to manufacturer’s instructions. Bisulfite treatment and further sample processing was carried out at the Mutation Analysis Facility, Karolinska Institutet, Sweden. Primers (Table 1) were designed and validated using the EpiDesigner web resource and the RseqMeth and MassArray packages in R. Two *IGF2AS* CpG units with single nucleotide polymorphisms were excluded from the analysis. One *IGF2_05* CpG unit was excluded from the analysis because of unreliable data indicated by low peaks and low signal-to-noise ratios. Thus, we finally examined four *IGF2AS* and six *IGF2_05* CpG units in this study. Quality control of the data was performed using EpiTYPER and MassArray R-script software. Assessment of primer-dimer formation and bisulfite conversion efficiency was made with MassArray R-script and indicated overall good quality data. Samples with low signal, low signal-to-noise ratio and low probability of accurate data signaled by EpiTYPER software were eliminated. All samples were measured in duplicate. Samples with a duplicate difference of greater than 20% in methylation level were considered unreliable and excluded from the analysis. Altogether seven measurements were excluded. We used means of each duplicate as the methylation level of that CpG unit in the analyses.

**Table 1:** *IGF2AS* and *IGF2_05* amplicons.

Amplicon	Primers	Amplicon length	Coordinates in build GRCh37/hg19	Coordinates in build NCBI36/hg18
*IGF2AS*	F:aggaagagTGGATAGGAGATTGAGGAGAAA	338	chr11:2169459-2169796	chr11:2126035-2126372
	R:cagtaatacgactcactatagggagaaggctAAACCCCAACAAAAACCACT			
*IGF2_05*	F:aggaagagagGAAGGGGTTGGTTAGTAGGTGTTTGT	276	chr11:2154463-2154738	chr11:2111039-2111314
	R:agtaatacgactcactatagggagaaggctCCTAAACCCCTTTCCCACTCTCTAA			

### Statistical analyses

Statistical analyses were performed using IBM SPSS Statistics 19.0 (Chicago, IL). To compare the baseline characteristics we used the t test for continuous and the χ^2^ test for categorical variables.

To compare the methylation fraction at each *IGF2AS* and *IGF2_05* CpG units, we used linear regression analysis with Bonferroni correction for multiple comparisons. We adjusted for covariates in different models; for plate number (dummy-coded), sex, and age in model 1, and for plate number, sex, age, height, BMI, mother’s smoking during pregnancy [25], mother’s age at birth, father’s age at birth, mother’s BMI before pregnancy [26], and highest educational attainment of either parent in model 2 (full adjustment). In an additional model we also adjusted for folic acid use of the subject [27]. Furthermore, in a subgroup with available data we adjusted for lean body mass instead of height and BMI.

Some of the VLBW subjects’ birth weight was equal to or less than 1500 g (inclusion criterion) because of being just born remarkably early, but appropriate for gestational age (AGA; birth weight ≥ –2SD) [23]. Other VLBW subjects were born SGA with less prematurity and had a birth weight of ≤1500 g caused by poor intrauterine growth. We thus also examined the methylation fractions between AGA and SGA VLBW subjects.

## Results

Characteristics of the study participants are presented in Table 2. The VLBW participants were born at the mean gestational week of 29.2 weeks (SD 2.2 weeks), and controls at 40.1 weeks (SD 1.2 weeks). Mean birth weight of the VLBW subjects was 1117 g (SD 220 g), and of the controls 3602 g (SD 478 g). Of the VLBW subjects, 53 (34%) were born SGA.

**Table 2:** Characteristics of the study participants.

Characteristic	VLBW (n = 158)	Controls (n = 161)	*P* a	Missing values (VLBW/controls)
Women, n (%)	91 (58)	96 (60)	0.9	0/0
Men, n (%)	67 (42)	65 (40)	0.8	0/0
Birth				
Gestational age, mean (SD), wk	29.2 (2.2)	40.1 (1.2)	<0.0001	0/0
Birth weight, mean (SD), g	1117 (220)	3602 (478)	<0.0001	0/0
Birth weight SDS, mean (SD), SDS	–1.29 (1.53)	0.06 (1.04)	<0.0001	0/0
SGA, n (%)	53 (34)	0		0/0
Parental				
Mother’s age	29.8 (4.8)	29.9 (5.1)	0.1	0/1
Father’s age	31.6 (5.6)	32.0 (6.0)	0.4	1/3
Mother’s BMI before pregnancy	22.2 (3.7)	22.3 (3.5)	0.2	27/3
Mother’s smoking during pregnancy	29 (18)	26 (16)	0.7	0/1
Highest education of either parent, n (%)				2/1
Elementary	16 (10)	9 (6)	0.2	
High school	34 (22)	28 (17)	0.4	
Intermediate	64 (41)	53 (33)	0.3	
University	42 (27)	70 (43)	0.03	
Current				
Age, mean (SD), y	22.4 (2.1)	22.5 (2.2)	0.1	0/0
Height, mean (SD), cm				
Women	162.0 (7.8)	167.4 (6.8)	<0.0001	0/0
Men	174.2 (7.6)	180.5 (6.5)	<0.0001	0/0
BMI, mean (SD), kg/m^2^				
Women	22.1 (3.8)	22.8 (3.6)	0.2	0/0
Men	22.0 (3.7)	23.3 (3.3)	0.03	0/0
Lean body mass, mean (SD), kg				
Women	38.9 (5.7)	43.0 (5.5)	<0.0001	5/19
Men	53.4 (8.1)	61.6 (8.0)	<0.0001	7/15

VLBW was birth weight ≤1500 g; SGA was birth weight less than –2SD.

a A t test for continuous and chi square test for categorical variables.

Table 3 shows the average methylation percent (%) and standard deviations (SDs) at each CpG unit of the *IGF2AS* and *IGF2_05* DMR amplicons in VLBW and in control subjects, as well as the numbers of measurements that were considered reliable.

**Table 3:** Mean (SD) methylation % at different *IGF2* CpG units in VLBW and control subjects.

*IGF2* CpGs	VLBW (n = 158)	Controls (n = 161)	Missing values (VLBW/controls)
	Mean % (SD)	Mean % (SD)	
*IGF2AS*			
CpG3	55.6 (0.04)	57.4 (0.05)	1/0
CpG4	60.4 (0.07)	60.9 (0.06)	1/2
CpG67	39.9 (0.04)	40.7 (0.04)	0/2
CpG8	51.8 (0.05)	52.8 (0.04)	1/2
*IGF2_05*			
CpG12	68.5 (0.05)	68.4 (0.05)	0/0
CpG34	67.4 (0.05)	67.1 (0.05)	0/0
CpG6	50.6 (0.04)	50.7 (0.04)	0/0
CpG7	56.3 (0.05)	56.4 (0.04)	0/1
CpG8	55.4 (0.04)	55.3 (0.04)	0/0
CpG91011	53.1 (0.05)	52.7 (0.05)	0/0

VLBW was birth weight ≤1500 g.

### Differences in IGF2 methylation between VLBW and control subjects

Mean differences and 95% confidence intervals (CIs) in methylation fractions at *IGF2AS* and *IGF2_05* CpG units between VLBW and control subjects, obtained by linear regression and full adjustment (model 2) are presented in Table 4. P-values for model 1 as well as unadjusted P-values are also shown. Males and females were analyzed together; sex interaction was not significant (P values ≥ 0.1). We found that the methylation at all *IGF2AS* CpG units tended to be lower in VLBW than in control subjects, but the difference was significant only at *IGF2AS* CpG3 [–0.017 (95% CI –0.028, –0.005), P = 0.004, full adjustment]. As there were multiple comparisons (10 CpG units), we carried out Bonferroni correction, in which case this difference between VLBW and term remained significant. At *IGF2_05* CpG unit methylation fractions were similar in both groups.

**Table 4:** Differences in methylation fractions at different *IGF2* CpG units between VLBW and controls by linear regression.

	Model 2		Model 1	Unadjusted
	Difference between VLBW and controls (95% CI)	*P*	*P*	*P*
*IGF2AS*				
CpG3	–0.017 (–0.028, –0.005)	0.004	0.0005	0.0004
CpG4	–0.010 (–0.026, 0.007)	0.248	0.527	0.505
CpG67	–0.008 (–0.017, 0.001)	0.099	0.051	0.057
CpG8	–0.008 (–0.020, 0.004)	0.178	0.053	0.066
*IGF2_05*				
CpG12	0.004 (–0.008, 0.017)	0.511	0.846	0.860
CpG34	0.005 (–0.008, 0.018)	0.435	0.577	0.595
CpG6	–0.002 (–0.012, 0.009)	0.776	0.708	0.733
CpG7	0.001 (–0.011, 0.012)	0.933	0.745	0.765
CpG8	0.003 (–0.008, 0.013)	0.610	0.705	0.698
CpG91011	0.008 (–0.004, 0.021)	0.174	0.446	0.422

VLBW was birth weight ≤1500 g.

Model 1; adjusted for plate n:o, sex, age.

Model 2; adjusted for plate n:o, sex, age, height, body mass index, mother’s smoking during pregnancy, mother’s age, father’s age, mother’s body mass index before pregnancy, and highest education of either parent.

Adjustment for folic acid use did not change the results (data not shown). In a subgroup with available data on body composition and adjustment for lean body mass instead of BMI and height, *IGF2AS* CpG3’s lower methylation fraction in VLBW compared to control subjects remained significant (P = 0.006). Other results also remained the same. Mode of delivery was not associated with methylation levels.

Of the covariates, age of the subject had a negative effect on methylation at *IGF2AS* CpG8 [–0.003 (95% CI –0.006, –0.001), P = 0.020], and mother’s smoking during pregnancy on *IGF2_05* CpG7 [–0.019 (95% CI –0.034, –0.004), P = 0.014] and *IGF2_05* CpG91011 [–0.017 (95%CI –0.032, –0.002), P = 0.031]. Other covariates did not have statistically significant effects on methylation fr actions (Table S1).

We also tested whether adult size-related characteristics or glucose values in the 2-hour OGTT were associated with *IGF2* methylation levels. Final height or adult BMI were not associated with methylation levels at *IGF2* CpG units in our study. Neither were methylation levels associated with 2-hour glucose levels, but there was a statistically significant association between fasting glucose and methylation level at *IGF2_05* CpG 12 (B = 1.004, 95%CI: 0.017 to 1.992, P =  0.046) and *IGF2_05* CpG 34 (B = 0.957, 95%CI: 0.012 to 1.903, P = 0.047).

### Differences in IGF2 methylation between VLBW subjects born SGA and those born AGA

In analyses within the VLBW group between the VLBW subjects born SGA and AGA, there were no significant differences in the methylation fractions at any *IGF2AS* or *IGF2_05* CpG units. (Table 5). The difference in *IGF2AS* CpG3 unit methylation was similarly different between VLBW-SGA and controls [–0.020 (95% CI: –0.037, –0.003), P = 0.022], as well as between VLBW-AGA and controls [–0.018 (95% CI: –0.031, –0.005), P = 0.007]. A history of perinatal infection, bronchopulmonary dysplasia, or maternal preeclampsia was not associated with methylation levels. These were also similar in VLBW subjects from multiple and those from singleton pregnancies (data not shown). Within the VLBW group methylation levels were not associated with birth weight or gestational age.

**Table 5:** Differences in methylation fractions at different *IGF2* CpG units between VLBW subjects born SGA and VLBW subjects born AGA by linear regression.

	Difference between VLBW-SGA and VLBW-AGA (95% CI)	*P*
*IGF2AS*		
IGF2_AS_CpG3	0.002 (–0.014, 0.019)	0.801
IGF2_AS_CpG4	0.002 (–0.022, 0.026)	0.125
IGF2_AS_CpG67	0.001 (–0.013, 0.015)	0.174
IGF2_AS_CpG8	0.001 (–0.017, 0.018)	0.953
*IGF2_05*		
IGF2_05_CpG12	–0.009 (–0.027, 0.010)	0.354
IGF2_05_CpG34	–0.011 (–0.029, 0.008)	0.253
IGF2_05_CpG6	–0.004 (–0.020, 0.012)	0.640
IGF2_05_CpG7	–0.004 (–0.022, 0.015)	0.685
IGF2_05_CpG8	–0.009 (–0.025, 0.007)	0.268
IGF2_05_CpG91011	–0.007 (–0.025, 0.011)	0.459

VLBW was birth weight ≤1500 g; SGA was birth weight less than –2SD; AGA was birth weigh equal to or more than 2SD.

## Discussion

Imprinted genes are epigenetically labile to early environmental influences [18]. These mitotically heritable marks may serve as archives of early exposure and may offer a fascinating mechanistic explanation to the concept of developmental origins of adult disease. The best characterized imprinted domain is *IGF2* that plays a key role in fetal growth, and alterations of methylation are present in fetal growth restriction [19] and in Beckwidth-Wiedemann and Silver-Russell syndromes [28], [29]. In this study, we investigated two DMRs at the *IGF2* locus in young adults born preterm at VLBW, who have previously been shown to have elevated levels of risk factors for CVDs in adulthood [10], [12]. We found that VLBW subjects have a significantly lower methylation level at one *IGF2AS* DMR CpG than their term-born peers. The finding survived Bonferroni correction of multiple comparisons and was not explained by potential confounding factors.

Although VLBW birth and *in utero* severe caloric restriction are not exactly similar early adverse events, our finding of 2% lower methylation in VLBW adults parallels that in the Dutch famine study, which showed that people who had been exposed to very early nutritional deprivation and had later increased incidence of chronic diseases had a 5% lower *IGF2* methylation level – measured decades after the exposure – at the same CpG sites that we measured [16]. Another evidence for epigenetic mechanisms influencing late-onset conditions comes from another study in which hypermethylation at some imprinted gene loci sensitive to prenatal nutrition were associated with myocardial infarction [30]. Recent studies have shown associations between gestational age at birth and methylation at patterns in newborns at several genes [31], [32]. We are not aware of other reports on DNA methylation patterns in subjects born preterm at VLBW. Although one study has reported severe hypomethylation at *IGF2/H19* locus in a term patient with birth weight less than -3SDS [33], another larger study on subjects born SGA did not reveal an association between *IGF2* methylation levels and SGA birth [34]. Accordingly, we did not find any difference in *IGF2* methylation between those VLBW subjects that were born SGA and those born AGA. Etiologies of SGA births are multiple and there are potentially many different epigenetically regulated loci [35], [36], as well as non-epigenetic factors [17] that lead to later health outcomes in these subjects. Other perinatal characteristics, such as infection, bronchopulmonary dysplasia, or preeclampsia, were unrelated to methylation levels. However, we suppose our subject population is not large enough to permit us to properly assess these effects. In addition, it has only limited power to assess associations with adult phenotypic characteristics.

It is not possible to assure here that the altered methylation in *IGF2* is not a primary defect that is responsible for the premature birth at VLBW. If the methylation change is secondary to VLBW birth, we can not be sure either that the 2% lower *IGF2AS* CpG3 methylation can have any clinical relevance. Whether such a minor reduction in methylation can have functional consequence, such as downregulation of *IGF2* function, is not known. However, methylation differences may be more significant in other tissues than the white blood cells from whole blood samples that we studied here [37], [38]. Analysis of methylation in, e.g., fat and muscle would give more detailed data for methylation associated with VLBW birth, but other tissues than blood were not available from our subjects. In addition to different tissues, methylation levels also vary across different blood cell types [38]. Thus, the methylation levels studied could be affected by differential cell count in whole blood samples. Using a publicly available database [39], we checked the methylation levels for available CpGs within 1 kb from our amplicons, and did not find major methylation differences between blood cell types that would have affected the interpretation of our results.

A clear advantage of this study is that the study population was exceptionally well characterized allowing for, e.g., adjustment for most important potential confounders that could affect *IGF2* methylation levels. It has been stated that infants born to smokers have higher methylation at the *IGF2* than those born to non-smokers [25]. Moreover, mother’s obesity before pregnancy may influence *IGF2* methylation and gene expression [26]. Although we observed a significant effect of mother’s smoking during pregnancy on methylation at some CpG units neither this nor any other confounding factor explained the differences in the *IGF2AS* CpG3 methylation fraction between VLBW and control subjects. We did not adjust for mode of delivery, since the reasons for cesarean section are different in preterm and term pregnancies. In our study, mode of delivery was not associated with methylation levels. In vitro fertilization has been reported to affect methylation patterns [40], but it was not used in the 1970s. Ethnicity could also have an influence on methylation patterns, but our study participants were almost exclusively of Finnish origin. Folic acid supplementation that has been suggested to increase the risk of adult chronic diseases has been reported to be associated with methylation levels [41]. We adjusted for folic acid use, but it did not change the results.

A minor disadvantage in our study is the number of study participants which was relatively small. Moreover, our study participants may not be representative of the original VLBW cohort, but this would only be expected to introduce bias if the association between VLBW birth and *IGF2* methylation was different among participants and non-participants. This cannot be excluded.

## Conclusions

In conclusion, we observed a significant difference in the methylation fraction at one *IGF2AS* DMR CpG site between preterm-born VLBW young adults with elevated levels of CVD risk factors in comparison to controls born at term. More data are needed to indicate whether *IGF2* methylation patterns can be causal for VLBW subjects’ higher levels of risk factors of cardiovascular disease and type 2 diabetes.

## Supporting Information

Table S1 **Differences in methylation fractions at different** ***IGF2AS*** **and** ***IGF2_05*** **CpG units between VLBW and control subjects by linear regression, and effects of covariates [B, 95% confidence interval (CI), and P] adjusted for in the model.**
(RTF)
