# Supplementary material for: Altered Methylation of IGF2 Locus 20 Years after Preterm Birth at Very Low Birth Weight
Source: PLoS One. 2013 Jun 19;8(6):e67379. doi: 10.1371/journal.pone.0067379 (PMC3686716; doi:10.1371/journal.pone.0067379)
Supplement: Table S1 — Differences in methylation fractions at different IGF2AS and IGF2_05 CpG units between VLBW and control subjects by linear regression, and effects of covariates [B, 95% confidence interval (CI), and P] adjusted for in the model. (RTF) [file pone.0067379.s001.rtf]

	B (95% CI)	P	
IGF2AS CpG3	-0.017 (-0.028, -0.005)	0.004	
plate n:o 	0.002 (-0.009, 0.013)	0.766	
sex	-0.008 (-0.023, 0.006)	0.267	
age 	0.001 (-0.002, 0.003)	0.682	
height	0.00005 (-0.001, 0.001)	0.904	
BMI 	0.000 (-0.002, 0.001)	0.653	
mother's smoking	-0.005 (-0.019, 0.010)	0.530	
mother's age	0.001 (-0.001, 0.002)	0.477	
father's age	0.000 (-0.001, 0.001)	0.744	
mother's BMI	0.001 (-0.001, 0.002)	0.349	
parental education	0.001 (-0.005, 0.007)	0.767	
IGF2AS CpG4	-0.010 (-0.026, 0.007)	0.248	
plate n:o 	0.003 (-0.012, 0.018)	0.695	
sex	-0.012 (-0.033, 0.008)	0.235	
age 	-0.001 (-0.005, 0.002)	0.474	
height	0.00001 (-0.001, 0.001)	0.983	
BMI 	-0.002 (-0.004, 0.000)	0.112	
mother's smoking	-0.011 (-0.031, 0.010)	0.307	
mother's age	-0.001 (-0.003, 0.001)	0.485	
father's age	-0.00001 (-0.002, 0.002)	0.991	
mother's BMI	0.001 (-0.002, 0.003)	0.602	
parental education	-0.002 (-0.011, 0.006)	0.625	
IGF2AS CpG67	-0.008 (-0.017, 0.001)	0.099	
plate n:o 	-0.003 (-0.012, 0.006)	0.526	
sex	-0.001 (-0.013, 0.010)	0.803	
age 	-0.001 (-0.003, 0.001)	0.257	
height	0.000 (-0.001, 0.000)	0.384	
BMI 	-0.001 (-0.002, 0.001)	0.255	
mother's smoking	0.002 (-0.010, 0.013)	0.774	
mother's age	0.000 (-0.001, 0.002)	0.613	
father's age	-0.001 (-0.002, 0.000)	0.202	
mother's BMI	0.001 (0.000, 0.002)	0.079	
parental education	0.002 (-0.003, 0.007)	0.425	
IGF2AS CpG8	-0.008 (-0.020, 0.004)	0.178	
plate n:o 	-0.003 (-0.014, 0.008)	0.650	
sex	-0.007 (-0.022, 0.007)	0.323	
age 	-0.003 (-0.006, -0.001)	0.020	
height	0.000 (-0.001, 0.001)	0.620	
BMI 	0.000 (-0.001, 0.002)	0.780	
mother's smoking	0.010 (-0.005, 0.024)	0.188	
mother's age	0.000 (-0.001, 0.002)	0.708	
father's age	0.000 (-0.002, 0.001)	0.485	
mother's BMI	0.001 (0.000, 0.003)	0.075	
parental education	0.004 (-0.002, 0.010)	0.240	
IGF2_05 CpG12	0.004 (-0.008, 0.017)	0.511	
plate n:o 	0.002 (-0.010, 0.013)	0.797	
sex	-0.002 (-0.018, 0.014)	0.797	
age 	0.001 (-0.002, 0.004)	0.404	
height	-0.00002 (-0.001, 0.001)	0.955	
BMI 	0.001 (-0.001, 0.002)	0.506	
mother's smoking	-0.006 (-0.022, 0.009)	0.417	
mother's age	-0.001 (-0.003, 0.000)	0.087	
father's age	0.001 (0.000, 0.002)	0.157	
mother's BMI	0.000 (-0.002, 0.001)	0.696	
parental education	0.000 (-0.007, 0.006)	0.881	
IGF2_05 CpG34	0.005 (-0.008, 0.018)	0.435	
plate n:o 	0.007 (-0.005, 0.019)	0.259	
sex	-0.001 (-0.017, 0.015)	0.880	
age 	0.002 (-0.001, 0.005)	0.266	
height	-0.00008 (-0.001, 0.001)	0.847	
BMI 	0.001 (-0.001, 0.002)	0.505	
mother's smoking	-0.006 (-0.023, 0.010)	0.445	
mother's age	-0.001 (-0.003, 0.001)	0.250	
father's age	0.001 (-0.001, 0.002)	0.288	
mother's BMI	-0.001 (-0.002, 0.001)	0.579	
parental education	0.003 (-0.004, 0.010)	0.419	
IGF2_05 CpG6	-0.012 (-0.012, 0.009)	0.776	
plate n:o 	-0.012 (-0.012, 0.008)	0.647	
sex	-0.015 (-0.015, 0.011)	0.755	
age 	-0.003 (-0.003, 0.002)	0.971	
height	-0.001 (-0.001, 0.001)	0.778	
BMI 	-0.002 (-0.002, 0.001)	0.658	
mother's smoking	-0.023 (-0.023, 0.004)	0.162	
mother's age	-0.002 (-0.002, 0.000)	0.193	
father's age	-0.001 (-0.001, 0.001)	0.731	
mother's BMI	-0.001 (-0.001, 0.002)	0.904	
parental education	-0.004 (-0.004, 0.008)	0.477	
IGF2_05 CpG7	0.001 (-0.011, 0.012)	0.933	
plate n:o 	-0.006 (-0.017, 0.005)	0.306	
sex	-0.002 (-0.017, 0.013)	0.752	
age 	0.001 (-0.002, 0.004)	0.526	
height	0.000 (-0.001, 0.001)	0.715	
BMI 	0.000 (-0.001, 0.002)	0.622	
mother's smoking	-0.019 (-0.034, -0.004)	0.014	
mother's age	-0.001 (-0.003, 0.000)	0.107	
father's age	0.000 (-0.001, 0.001)	0.809	
mother's BMI	0.001 (-0.001, 0.002)	0.414	
parental education	0.001 (-0.006, 0.007)	0.852	
IGF2_05 CpG8	0.556 (-0.008, 0.013)	0.610	
plate n:o 	0.003 (-0.006, 0.014)	0.409	
sex	0.004 (-0.017, 0.010)	0.624	
age 	-0.003 (-0.003, 0.002)	0.985	
height	-0.00002 (-0.001, 0.001)	0.965	
BMI 	0.00002 (-0.002, 0.001)	0.769	
mother's smoking	0.000 (-0.025, 0.002)	0.083	
mother's age	-0.012 (-0.002, 0.001)	0.237	
father's age	-0.001 (-0.001, 0.002)	0.472	
mother's BMI	0.000 (-0.001, 0.002)	0.848	
parental education	0.000 (-0.003, 0.008)	0.435	
IGF2_05 CpG91011	0.008 (-0.004, 0.021)	0.174	
plate n:o 	-0.003 (-0.014, 0.009)	0.660	
sex	-0.004 (-0.019, 0.011)	0.593	
age 	0.000 (-0.002, 0.003)	0.791	
height	0.000 (-0.001, 0.001)	0.775	
BMI 	0.00004 (-0.002, 0.002)	0.964	
mother's smoking	-0.017 (-0.032, -0.002)	0.031	
mother's age	-0.002 (-0.003, 0.000)	0.056	
father's age	0.001 (-0.001, 0.002)	0.225	
mother's BMI	0.001 (-0.001, 0.002)	0.515	
parental education	0.001 (-0.005, 0.008)	0.695	

Supplementary table. Differences in methylation fractions at different IGF2AS and IGF2_05 CpG sites between VLBW and control subjects by linear regression, and effects of covariates [B, 95% confidence interval (CI), and P] adjusted for in the model.
